# Pisachini planthoppers of Vietnam: new records of *Pisacha* and a new *Goniopsarites* species from Central Vietnam (Hemiptera, Fulgoromorpha, Nogodinidae)

**DOI:** 10.3897/zookeys.1193.114957

**Published:** 2024-03-06

**Authors:** Jérôme Constant, Thai-Hong Pham, Cuong Viet Canh Le, Trung Thanh Vu, Hoai Thu Thi Nguyen, Hai Nam Tran

**Affiliations:** 1 Royal Belgian Institute of Natural Sciences, O.D. Taxonomy & Phylogeny – Entomology, Vautier street 29, B-1000 Brussels, Belgium Royal Belgian Institute of Natural Sciences Brussels Belgium; 2 Mientrung Institute for Scientific Research, Vietnam National Museum of Nature, VAST, 321 Huynh Thuc Khang, Hue, Vietnam Graduate School of Science and Technology, Vietnam Academy of Science and Technology Hanoi Vietnam; 3 Graduate School of Science and Technology, Vietnam Academy of Science and Technology, 18 Hoang Quoc Viet, Hanoi, Vietnam Vietnam National Museum of Nature Hue Vietnam; 4 Vietnam National Museum of Nature, Vietnam Academy of Science and Technology (VAST), 18 Hoang Quoc Viet, Hanoi, Vietnam Vietnam National Museum of Nature, Vietnam Academy of Science and Technology Hanoi Vietnam; 5 Department of Biology, Hanoi National University of Education, 136 Xuan Thuy, Cau Giay, Hanoi, Vietnam Hanoi National University of Education Hanoi Vietnam

**Keywords:** Bach Ma National Park, biodiversity, Fulgoroidea, Indochina, Phong Dien District

## Abstract

Two planthopper species of the family Nogodinidae are added to the fauna of Vietnam, both from two localities in Thua Thien-Hue Province: Bach Ma National Park and Phong Dien District. The first species belongs to *Goniopsarites* Meng, Wang & Wang, 2014, *G.mientrunganus* Constant & Pham, **sp. nov.**, and the second belongs to *Pisacha* Distant, 1906, *P.yinggensis* Meng, Wang & Wang, 2014. *Pisachayinggensis* was previously recorded from Hainan Island, China. These new records greatly extend the distribution of both genera, which were known from southern China, Hainan and North Vietnam, to the south, reaching the mid area of Central Vietnam. Sexual dimorphism is reported in *P.yinggensis* for the first time. Illustrations of habitus and male terminalia of the new species are given as well as distribution maps and photographs of live specimens and their habitat. The family Nogodinidae now comprises nine species in Vietnam, with three of them present in Bach Ma National Park.

## ﻿Introduction

The family Nogodinidae Melichar, 1898 is distributed worldwide in the tropics and subtropics and contains 378 species in 99 genera, including 11 species in Vietnam (Bourgoin 2023). Of these 11 species, five were described in the last 10 years (Constant and Pham 2014; Gnezdilov and Constant 2014; Meng et al. 2014; Constant and Pham 2016). Within the subfamily Nogodininae Melichar, 1898, the tribe Pisachini Fennah, 1978 was recently reviewed by Meng et al. (2014) and currently counts 10 species in three genera distributed in Southeast Asia (Constant and Pham 2016; Bourgoin 2023).

Study of the recent material of Nogodinidae in the collections of the Vietnam National Museum of Nature and Royal Belgian Institute of Natural Sciences revealed two species of Pisachini from Central Vietnam, which are new to the fauna of the country, including a species of *Goniopsarites* Meng, Wang & Wang, 2014 new to science.

The present paper describes this new species and provides the first Vietnamese records of *Pisachayinggensis* Meng, Wang & Wang, 2014 as a new contribution to the Vietnamese nogodinid fauna.

## ﻿Material and methods

The terminalia were extracted after soaking the abdomen overnight in a 10% solution of potassium hydroxide (KOH) at room temperature. The pygofer was separated from the abdomen, thoroughly rinsed in 70% ethanol, and the aedeagus dissected with a needle blade for examination. The whole was then placed in glycerin for preservation in a tube attached to the pin of the corresponding specimen. The hind wing was mounted, glued on a white cardboard rectangle with white glue, and the cardboard attached to the pin of the specimen. Photographs of collection specimens were taken with a Leica EZ4W stereomicroscope, stacked with CombineZ software, and optimized with Adobe Photoshop software; photographs from the field were taken with an Olympus Tough 6 camera. The maps were produced with SimpleMappr (Shorthouse 2010) and include records available from Meng et al. (2014) and Constant and Pham (2016). The external morphological terminology follows O’Brien and Wilson (1985), the wing venation terminology follows Bourgoin et al. (2015) and, for the male terminalia, Bourgoin and Huang (1990). The metatibiotarsal formula gives the number of spines on (side of metatibia) apex of metatibia / apex of first metatarsomere / apex of second metatarsomere.

The measurements were taken as by Constant (2004) and the following acronyms are used:

**BB** maximum breadth of the body;

**BF** maximum breadth of the fron;

**BTg** maximum breadth of the tegmen;

**BV** maximum breadth of the vertex;

**LF** length of the frons at median line;

**LT** total length (apex of head to apex of tegmina);

**LTg** length of the tegmen;

**LV** length of the vertex at median line.

Acronyms used for the collections:

**RBINS**Royal Belgian Institute of Natural Sciences, Brussels, Belgium;

**VNMN**Vietnam National Museum of Nature, Hanoi, Vietnam.

Other abbreviations:

**CCRR** Centre for Conservation of Vietnam Natural Resources and Rescue of Animals and Plants.

## ﻿Taxonomy

### ﻿Family Nogodinidae Melichar, 1898

**Subfamily Nogodininae Melichar, 1898**

**Tribe Pisachini Fennah, 1978**

#### ﻿Genus Goniopsarites

Taxon classificationAnimaliaHemipteraNogodinidae

Meng, Wang & Wang, 2014

40CE3D7C-AB33-5BBD-8E95-51F2C2F79E82

Goniopsarites Meng et al., 2014: 80, figs 1–27.

##### Type species.

*Goniopsaritesfronticonvexus* Meng, Wang & Wang, 2014, by original designation.

##### Distribution.

Southern China (Guangdong, Hainan); North and Central Vietnam.

##### Species included.

*G.fronticonvexus* Meng, Wang & Wang, 2014 – China: Hainan and Guangdong.

*G.mientrunganus* Constant & Pham, sp. nov. – Vietnam: Bach Ma National Park and Phong Dien CCRR.

*G.tonkinensis* Constant & Pham, 2016 – Vietnam: Ba Be and Cuc Phuong national parks, and Me Linh Biodiversity Station.

### ﻿Key to the species of *Goniopsarites*

**Table:** 

1	Posterior processes of the periandrium (*ppp* – Fig. 2D) large and laminate, curved dorsocephalad in lateral view; posterodorsal process of the gonostyli bulging apically (*G* – Fig. 2A); basiventral lobe of the proximal half of the anal tube strongly developed in lateral view (*An* – Fig. 2A)	***Goniopsaritesmientrunganus* Constant & Pham, sp. nov.**
–	Posterior processes of the periandrium not large and laminate, but instead indistinct (Meng et al. 2014: fig. 19) or slender and curved posterodorsad (Constant and Pham 2016: fig. 4C); posterodorsal process of the gonostyli not bulging apically (Meng et al. 2014: fig. 18; Constant and Pham 2014: fig. 4A); basiventral lobe of the proximal half of the anal tube moderately developed in lateral view (Meng et al. 2014: fig. 18; Constant and Pham 2014: fig. 4A)	**2**
2	Anal tube strongly curved and very wide apically in lateral view (Constant and Pham 2016: fig. 4A) and with lateral margins strongly sinuate and narrowing towards base in dorsal view (Constant and Pham 2016: fig. 4B); apex of aedeagus more elongate and curved ventrally at apex (Constant and Pham 2016: fig. 4C); lateral processes of aedeagus strongly sinuate (Constant and Pham 2016, fig. 4C); posterior processes of the periandrium slender and curved posterodorsad (Constant and Pham 2016: fig. 4C)	***Goniopsaritestonkinensis* Constant & Pham, 2016**
–	Anal tube moderately curved, and roundly truncate apically in lateral view (Meng et al. 2014: fig. 18) and with lateral margins weakly sinuate and wider towards base in dorsal view (Meng et al. 2014: fig. 14); apex of aedeagus roundly truncate and not curved ventrally at apex (Meng et al. 2014: fig. 19); lateral processes of aedeagus regularly curved in lateral view (Meng et al. 2014: fig. 19); posterior processes of the periandrium indistinct (Meng et al. 2014: figs 19, 20)	***Goniopsaritesfronticonvexus* Meng, Wang & Wang, 2014**

#### ﻿ Goniopsarites mientrunganus

Taxon classificationAnimaliaHemipteraNogodinidae

Constant & Pham sp. nov.

81D7FFA7-4E0B-5432-A46C-66C3F695FA8F

https://zoobank.org/689317C8-D1E4-4526-81CC-E89B9C11E42B

Figs 1 , 2 , 3 , 4A

##### Type material.

***Holotype*** ♂, Vietnam • Thừa Thiên-Huế Province, Bach Ma National Park, near ranger station; 16°08'37"N, 107°49'36"E; 18 May 2023; alt. 300–600 m; J. Constant & L. Semeraro leg.; VNMN_E000.017.000.

***Paratypes***, Vietnam • 1 ♀; Thừa Thiên-Huế Province, Bach Ma National Park; 16°13'14"N, 107°53'10"E; 9 Mar. 2023; by net; V.T. Trung leg.; VNMN_ E000.017.001 • 1 ♂; Thừa Thiên-Huế Province, Bach Ma National Park, Pheasant trail; 16°13'38"N, 107°51'20"E; 10–20 May 2023; alt. 500–600 m; J. Constant & L. Semeraro leg.; I.G.: 34.640; RBINS • 2 ♂♂, 1 ♀; Thừa Thiên-Huế Province, Bach Ma National Park; low altitude; 16°13'05"N, 107°42'27"E; 17 May 2023; alt. 100–200 m; J. Constant & L. Semeraro leg.; I.G.: 34.640; RBINS • 1 ♀; Thừa Thiên-Huế Province; Phong Dien District, CCRR; 16°30'27"N, 107°16'05"E; 23 May 2023; alt. 350–400 m; J. Constant & L. Semeraro leg.; I.G.: 34.640; RBINS • 1 ♀; same collection data as for preceding; VNMN_E000.017.002.

##### Diagnosis.

The species is very close externally to *G.fronticonvexus* Meng, Wang & Wang, 2014 and *G.tonkinensis* Constant & Pham, 2016 but has slightly more elongate tegmina, 2.2 times as long as wide (1.9 times in *G.fronticonvexus*; 2.0 times in *G.tonkinensis*). These species are better separated by the male genitalia characters as follows.

*Goniopsaritesmientrunganus* Constant & Pham, sp. nov. can be separated from the other two species by **(1)** its large laminate posterior processes of the periandrium (*ppp* – Fig. 2D), which are absent in *G.fronticonvexus* and much more slender, curved the other way round, in *G.tonkinensis*; **(2)** the posterodorsal process of the gonostyli bulging apically (*G* – Fig. 2A), not bulging in both other species; and **(3)** the strongly developed basiventral lobe of the proximal half of the anal tube (*An* – Fig. 2A), clearly less developed in both other species.

From *G.tonkinensis*, *G.mientrunganus* Constant & Pham, sp. nov. also differs in **(1)** having the maximum width of the anal tube in the basal third (*An* – Fig. 2C), which is in the distal half of the anal tube in *G.tonkinensis* (the state of this character is not known for *G.fronticonvexus*); **(2)** in having a strongly developed dorsal laminate process along basal half of periandrium (*dlp* – Fig. 2D, F, G).

##### Description.

***Measurements and ratios***: LT: ♂ (*n* = 4): 11.3 mm (11. 1–11.7); ♀ (*n* = 4): 13.5 (13. 0–14.5). LTg/BTg = 2.2; LW/BW = 1.4; LV/BV = 8.5; LF/BF = 1.4.

**Figure 1.**
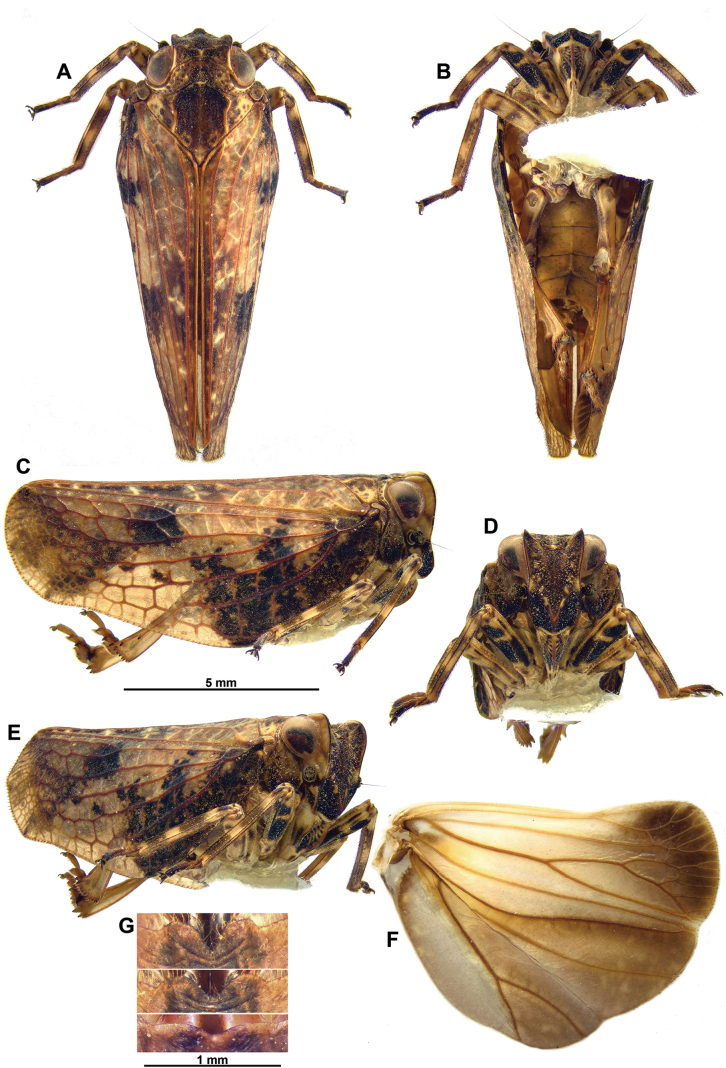
*Goniopsaritesmientrunganus* Constant & Pham, sp. nov. **A–F** holotype ♂ **A** habitus, dorsal **B** habitus, ventral **C** habitus, lateral **D** habitus, perpendicular view of frons **E** habitus, anterolateral **F** right hind wing **G** paratypes ♀♀, posterior margin of sternite VII.

***Head***: (Fig. 1A–E) vertex dark brown, with yellowish median and lateral carinae; concave with lateral margins carinate, and with anterior and posterior margins slightly carinate and rather strongly concave in dorsal view, resulting in a very narrow central portion. Frons varying from variegated brown and yellowish, darker under V-shaped carina, carina yellowish to reddish, to nearly completely black-brown, sometimes with yellowish markings around mid-height of lateral margin; narrow, well-defined, yellowish line above frontoclypeal suture; frons elongate, broader dorsally, concave with median carina on middle of disc and with a strong projection on ventral half marked by strong, V-shaped carina extending ventrad on clypeus; projection rounded in lateral view; lateral margins of frons carinate; frontoclypeal suture grooved and rounded ventrally. Genae largely variegated yellow-brown, dark brown under antennae, with pale yellowish spot along anterior margin. Clypeus yellowish, variegated with black-brown on sides towards apex, and with brown oblique lines on each side; clypeus elongate, narrower and shorter than frons, and showing a strong median carina; roundly convex in lateral view. Labium elongate and narrow, yellow-brown, slightly surpassing posterior coxae. Antennae black with scape ring-shaped; pedicel subglobose.

***Thorax***: (Fig. 1A, C–E) pronotum variegated yellow-brown, darker in middle portion, rather densely pitted, and with median carina weakly marked; lateral fields of prothorax coloured as pronotum, darker in ventral portion, widening ventrally and with ventral margin rounded. Mesonotum dark brown, with yellowish markings on sides and yellowish spot on scutellum; lateral fields moderately pitted; median carina obsolete; lateral carinae marked with yellowish, fusing anteriorly in a rounded carina parallel to posterior margin of pronotum. Tegulae brown.

***Tegmina***: (Fig. 1A–C) elongate, with costal margin broadly rounded on proximal half and rather strongly sinuate on posterior half; brown with large, irregular, black-brown marking on basal half, not extending on clavus; large, pale yellowish marking along costal margin in distal half; clavus with irregular, slightly darker markings on posterior half; hypocostal plate narrow, visible in proximal third; posterior margin obliquely rounded; clavus closed, extending to posterior angle of tegmen. Veins reddish brown, sometimes black in black areas.

***Hind wings***: (Fig. 1F) brown, darker towards posterocostal angle and with large basicostal yellow-brown area; well developed, with posterior margin trilobed; costal margin sinuate, with coupling apparatus at 2/3 of length.

***Legs***: (Fig. 1A–C) profemora black-brown, with narrow, pale yellow rings; mesofemora pale yellow, with brown rings; pro- and mesotibiae elongate and slender, with 6 rings alternatively pale yellow and brown, basal one brown, apical one yellow; metafemora yellow, with brown markings; metatibiae rather short, broadening towards apex, pale yellow, infuscate basally, with 2 strong lateral spines near apex. Tarsi brown, darker apically. Metatibiotarsal formula: (2) 10 / 2 (+7 on underside) / 2.

***Abdomen***: (Fig. 1B) pale yellowish brown.

**Figure 2.**
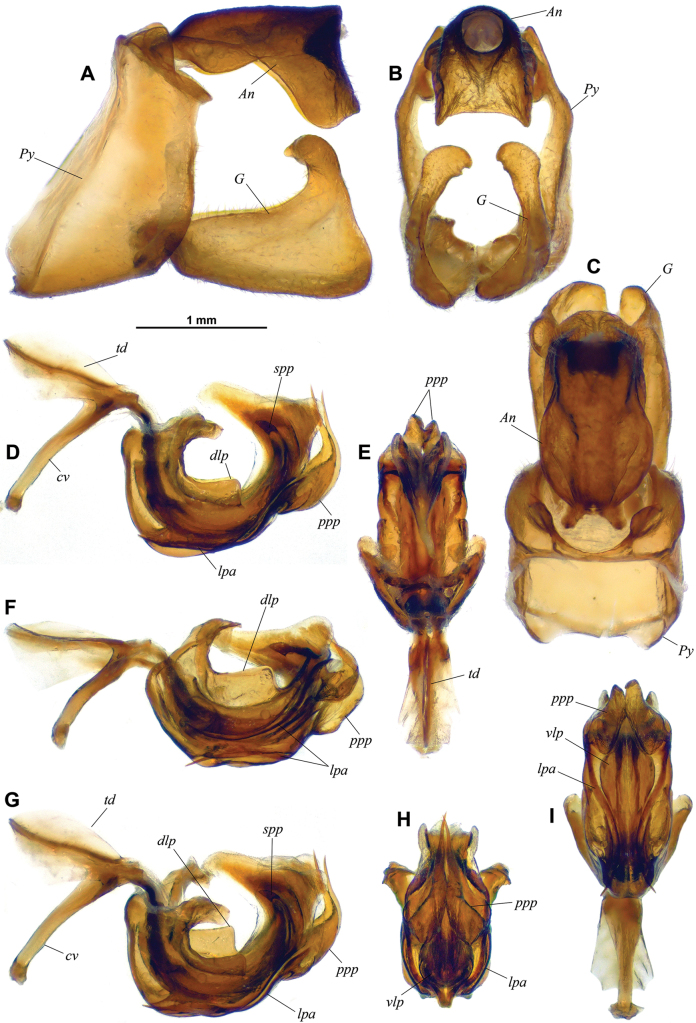
*Goniopsaritesmientrunganus* Constant & Pham, sp. nov., holotype ♂, terminalia **A–C** pygofer, gonostyli and anal tube **A** lateral **B** caudal **C** dorsal **D–I** aedeagus, phallobase and connective **D** left lateral **E** dorsal **F** lateroventral **G** laterodorsal **H** caudal **I** ventral. *cv* = connective; *dlp* = dorsal laminate process of periandrium; *lpa* = lateral process of aedeagus; *ppp* = posterior process of periandrium; *spp* = sclerotized process of phallus; *td* = tectiductus; *vlp* = ventral lobe of periandrium.

***Terminalia*** ♂: (Fig. 2) pygofer (*Py*) higher than long in lateral view, with anterior and posterior margins moderately sinuate; posterodorsal angles with dorsally developed, subtriangular laminate process, with posterodorsal angle right and rounded. Anal tube (*An*) massive, elongate, with lateral margins in dorsal view, widely rounded in proximal half, then sinuately tapering towards posterior, leaving widely rounded ventral margin visible on sides in distal half; 1.78 times as long in midline, as wide with maximum width at proximal 1/3 in dorsal view; basal half in lateral view with large ventral lobe; strongly curved ventrally at midlength with lateral margins strongly produced ventrally into broad lobe in distal half. Gonostyli (*G*) (Fig. 4A) elongate in lateral view, rounded apically and with strong process projecting dorsomesad at posterodorsal angle; ventral margin nearly straight and posterior margin weakly sinuate in lateral view; dorsal process slightly twisted internally, bulging in distal portion and with apex with blunt point directed anteromesad. Aedeagus (Fig. 2D–I) strongly curved in lateral view, with a pair of lateral, elongate, strongly sinuate processes (*lpa*) directed anteriorly, attached posteriorly at 2/3 of length; processes well visible and sinuate in ventral view. Periandrium posteriorly with a pair of large laminate processes (*ppp*), lobe-shaped and developed dorsally into sinuate, elongate, narrowly pointed processes directed mesodorsad; well-developed laminate process (*dlp*) running dorsally in basal half, abruptly terminated at right angle at midlength. Ventral lobe of periandrium (*vlp*) elongate, lanceolate in distal portion in ventral view, with sides rounded. Phallus membranous, large, with pair of curved, sclerotized processes (*spp*) directed cephalodorsad before apex; strongly, angularly projecting posterodorsad; apex narrowing, directed anteriorly, and curved ventrally. Connective (*cv*) with well-developed, elongate tectiductus (*td*) showing complete, broadly rounded dorsal crista.

##### Note.

The female genital structures were checked and found to be similar to those of *G.fronticonvexus*, as well as to these of *G.tonkinensis*, and, as it is the case in most nogodinids that we have observed so far, female genitalia do not help with species identification. The indentation in middle of the hind margin of sternum VII is always present but was found to vary in depth between the specimens of *G.mientrunganus* sp. nov. that we have in hand (Fig. 1G).

##### Etymology.

The species epithet *mientrunganus* refers to the region where the new species was discovered: Central Vietnam, “Miền Trung” in Vietnamese.

##### Biology.

**Figure 3.**
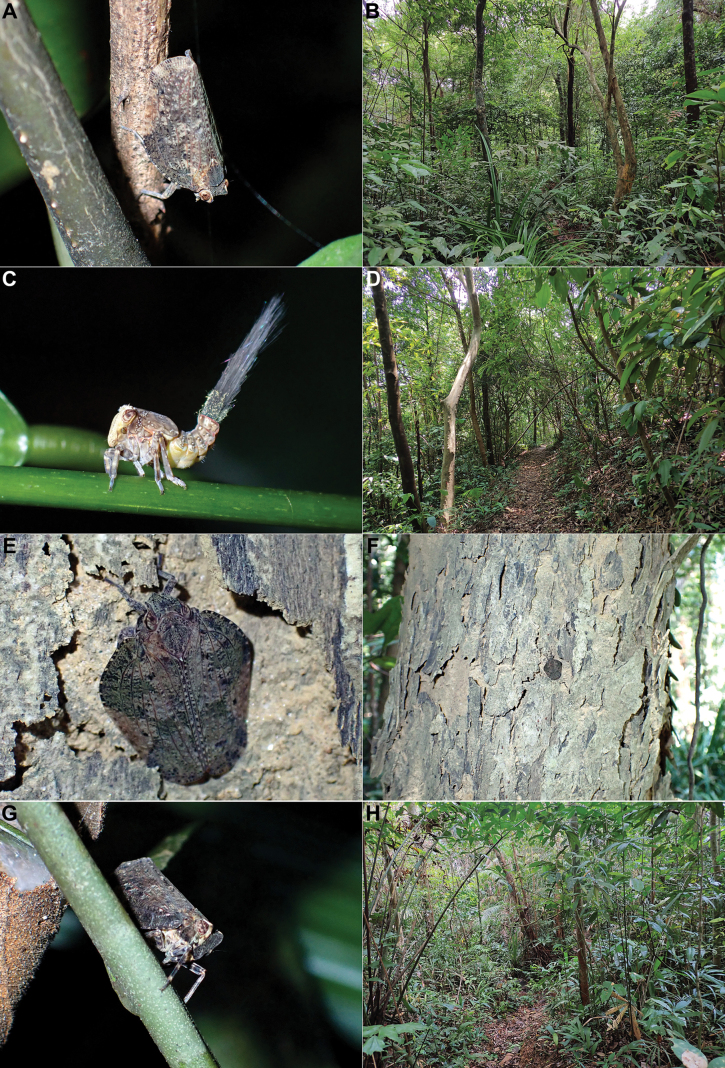
*Goniopsaritesmientrunganus* Constant & Pham, sp. nov., in nature and habitat **A–C** Bach Ma National Park, low altitude, 15 May 2023 **A** adult on stem **B** habitat **C** nymph **D–F** Bach Ma National Park, Pheasant Trail, 20 May 2023 **D** habitat **E** adult on tree trunk **F** idem, general view **G, H** Phong Dien District, CCRR, 23 May 2023 **G** adult on stem **H** habitat.

The specimens were found sitting on stems of bushes (Fig. 3A, C, G) or on tree trunks (Fig. 3E, F), in subtropical evergreen forest (Fig. 3B, D, H) at the junction of the Northern Vietnam lowland rain forests, Southern Vietnam lowland rain forests, and Southern Annamites montane rain forests ecoregions, at rather low altitude (150–600 m). Some nymphs (Fig. 3C) were observed in May 2023 together with adult specimens.

##### Distribution.

**Figure 4.**
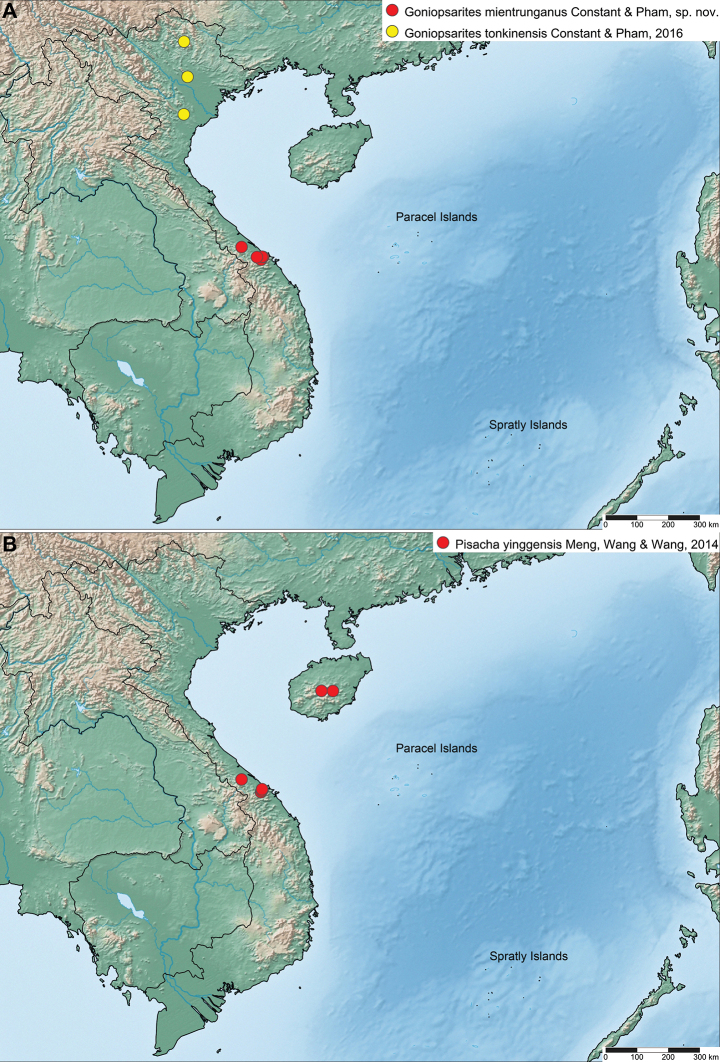
Distribution maps **A***Goniopsarites* species in Vietnam **B***Pisachayinggensis* Meng, Wang & Wang, 2014.

Vietnam, Thua Tinh-Hue Province, Bach Ma National Park, and Phong Dien district, CCRR (Fig. 4A).

#### ﻿Genus Pisacha

Taxon classificationAnimaliaHemipteraNogodinidae

Distant, 1906

91C920E8-CBC5-5A93-8B84-7AF5A844DA4E

Pisacha Distant, 1906: 391. Type species: Pisachanaga Distant, 1906, by original designation.
Soaemis Jacobi, 1915. Nomen nudum.
Soaemis Jacobi, 1916: 311. Type species: Soaemisencaustica Jacobi, 1916, by original designation. Synonymized by Ishihara (1965: 207).

##### Distribution.

Southern China (Chongqing, Guangdong, Hainan, Sichuan, Zhejiang); India (Assam); Taiwan; North and Central Vietnam.

##### Species included.

*P.baculiformis* Meng, Wang & Wang, 2014 – China: Zhejiang.

*P.balteiformis* Meng, Wang & Wang, 2014 – Vietnam: Ninh Binh Province.

*P.encaustica* (Jacobi, 1916) – Taiwan.

*P.falcata* Meng, Wang & Wang, 2014 – China: Chongqing, Sichuan.

*P.kwangsiensis* Chou & Lu, 1977 – China: Guangxi.

*P.naga* Distant, 1906 – India: Assam.

*P.yinggensis* Meng, Wang & Wang, 2014 – China: Hainan; Vietnam: Thua Thien-Hue Province.

#### ﻿ Pisacha yinggensis

Taxon classificationAnimaliaHemipteraNogodinidae

Meng, Wang & Wang, 2014

1C4B0DF6-EBFE-54EC-8578-EDEB9788F019

Figs 4B , 5

Pisacha yinggensis Meng, Wang & Wang, 2014: 93, figs 38–41, 56–69, 115, 120.

##### Material examined.

Vietnam • 1 ♂; Thừa Thiên-Huế Province; Phong Dien District, CCRR; 16°30'27"N, 107°16'05"E; 23 May 2023; alt. 350–400m; J. Constant & L. Semeraro leg.; I.G.: 34.640; RBINS • 1 ♂, 2 ♀♀; Thừa Thiên-Huế Province, Bach Ma National Park, near ranger station; 16°08'37"N, 107°49'36"E; 18 May 2023; alt. 300–600 m; J. Constant & L. Semeraro leg.; VNMN_ E000.017.003 • Thừa Thiên-Huế Province, Bach Ma National Park; 16°12'N, 107°52'E; 12–17 Jul. 2011; J. Constant & J. Bresseel leg.; I.G.: 31.933; RBINS • 1 ♀; Thừa Thiên-Huế Province, Bach Ma National Park; summit; 16°11'18"N, 107°50'56"E; 11–21 May 2023; alt. 1300–1400 m; J. Constant & L. Semeraro leg.; I.G.: 34.640; RBINS • 1 ♂, 1 ♀; Thừa Thiên-Huế Province, Bach Ma National Park, Pheasant trail; 16°13'38"N, 107°51'20"E; 10–20 May 2023; alt. 500–600 m; J. Constant & L. Semeraro leg.; I.G.: 34.640; RBINS • 2 ♀♀; same collection data as for preceding; VNMN_ E000.017.004 • 1 ♂; same collection data as for preceding; 29 Mar. 2021; Malaise trap; V.T. Trung leg.; VNMN_ E000.017.005 • 1 ♀; Thừa Thiên-Huế Province; Phong Dien District, CCRR; 16°30'27"N, 107°16'05"E; 23 May 2023; 350–400m; Trung T. Vu leg.; VNMN_ E000.017.006.

##### Notes.

**Figure 5.**
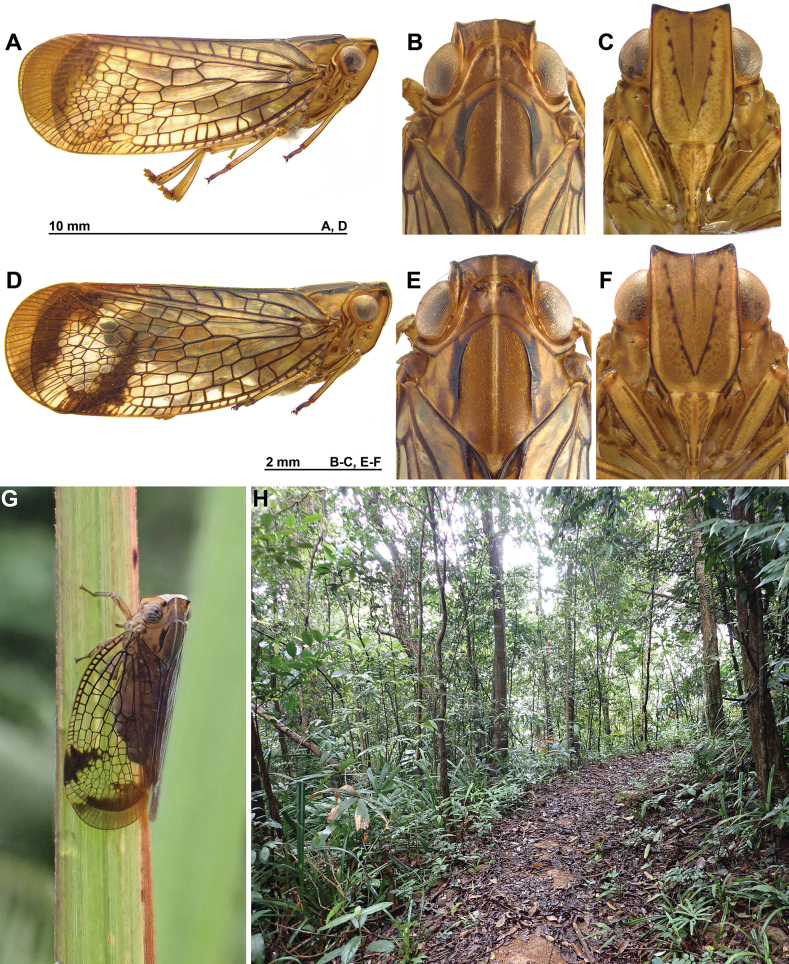
*Pisachayinggensis* Meng, Wang & Wang, 2014, from Bach Ma National Park, Pheasant Trail, 10–20 May 2023 **A–C** male **A** lateral **B** head and thorax, dorsal **C** frons, perpendicular **D–F** female **D** lateral **E** head and thorax, dorsal **F** frons, perpendicular **G** adult female in nature **H** habitat.

The specimens from Central Vietnam show obvious sexual dimorphism, with the tegmina of the females marked with a contrasting, incomplete, dark brown to black band at the distal 2/3 of its length, and a broad, oblique area of the same colour, or slightly paler, along the apical margin (Fig. 5D, G). These dark markings on the tegmina are weakly visible or absent in males (Fig. 5A). The markings of the head and thorax, however, are similar in both sexes (Fig. 5B, C, E, F).

##### Biology and distribution.

The specimens were found sitting on leaves or stems on the lower vegetation (Fig. 5G), in subtropical evergreen forest (Fig. 5H) in at the junction of Northern Vietnam lowland rain forests, Southern Vietnam lowland rain forests, and Southern Annamites montane rain forests ecoregions, at altitude from 300 to 1,400 m. In China, the species is recorded from the Hainan Island monsoon rain forests ecoregion.

The species was previously only recorded from Hainan Island, China (Meng et al. 2014). It is here recorded for the first time from the mainland, in Central Vietnam (Fig. 4B).

## ﻿Discussion

The present work adds two species of Nogodinidae to the fauna of Vietnam, including one species described as new. This leads to a total of 13 species for the country. As a comparison, 10 species are known from China (Bourgoin 2023). The new records also greatly extend the Vietnamese distribution of the genera *Goniopsarites* and *Pisacha* to the south but leave a gap of over 500 km without any records of these genera in the northern half of Central Vietnam.

In Phong Dien District, VNMN is conducting an ambitious project of forest restoration at the Centre for Conservation of Vietnam Natural Resources and Rescue of Animals and Plants, not far from the forest where *G.mientrunganus* Constant & Pham, sp. nov. and *P.yinggensis* were found. As already recently mentioned for a new species of Tropiduchidae, *Connelicitaphongdienensis* Constant & Pham, 2023, the return of such planthopper species in this area in the future would be a great indicator of a successful project (Constant et al. 2023).

## Supplementary Material

XML Treatment for Goniopsarites

XML Treatment for Goniopsarites mientrunganus

XML Treatment for Pisacha

XML Treatment for Pisacha yinggensis

## ﻿Additional information

### Author ORCIDs

Jérôme Constant https://orcid.org/0000-0003-0254-0863

Thai-Hong Pham https://orcid.org/0000-0002-4763-3679

Cuong Viet Canh Le https://orcid.org/0000-0002-1430-6305

Trung Thanh Vu https://orcid.org/0009-0002-8987-1672

Hoai Thu Thi Nguyen https://orcid.org/0009-0004-9130-2940

Hai Nam Tran https://orcid.org/0009-0003-1557-1666

### Data availability

All of the data that support the findings of this study are available in the main text.
